# To Each Stress Its Own Screen: A Cross-Sectional Survey of the Patterns of Stress and Various Screen Uses in Relation to Self-Admitted Screen Addiction

**DOI:** 10.2196/11485

**Published:** 2019-04-02

**Authors:** Najmeh Khalili-Mahani, Anna Smyrnova, Lisa Kakinami

**Affiliations:** 1 PERFORM Centre Design and Computation Arts Concordia University Montreal, QC Canada; 2 McGill Centre for Integrative Neuroscience Montreal Neurological Institute McGill University Montreal, QC Canada; 3 PERFORM Centre Department of Mathematics and Statistics Concordia University Montreal, QC Canada

**Keywords:** psychological stress, addictive behavior, coping behavior, social network, gaming, internet, communications, telemedicine, eHealth

## Abstract

**Background:**

The relationship between stress and screen addiction is often studied by exploring a single aspect of screen-related behavior in terms of maladaptive dependency or the risks associated with the content. Generally, little attention is given to the pattern of using different screens for different types of stressors, and variations arising from the subjective perception of stress and screen addiction are often neglected. Given that both addiction and stress are complex and multidimensional factors, we performed a multivariate analysis of the link between individual’s subjective perceptions of screen addiction, various types of stress, and the pattern of screen usage.

**Objective:**

Using the media-repertoires framework to study usage patterns, we explored (1) the relation between subjective and quantitative assessments of stress and screen addiction; and (2) differences in stress types in relation to subjective screen addiction and different types of needs for screens. We hypothesized that interindividual heterogeneity in screen-related behavior would reflect coping differences in dealing with different stressors.

**Methods:**

A multifactorial Web-based survey was conducted to gather data about screen-related behaviors (such as screen time, internet addiction, and salience of different types of screens and related activities), and different sources of stress (emotional states, perceptual risks, health problems, and general life domain satisfaction). We performed group comparisons based on whether participants reported themselves as addicted to internet and games (A1) or not (A0), and whether they had experienced a major life stress (S1) or not (S0).

**Results:**

Complete responses were obtained in 459 out of 654 survey responders, with the majority in the S1A0 (44.6%, 205/459) group, followed by S0A0 (25.9%, 119/459), S1A1 (19.8%, 91/459), and S0A1 (9.5%, 44/459). The S1A1 group was significantly different from S0A0 in all types of stress, internet overuse, and screen time (*P*<.001). Groups did not differ in rating screens important for short message service (SMS) or mail, searching information, shopping, and following the news, but a greater majority of A1 depended on screens for entertainment (χ^2^_3_=20.5; *P*<.001), gaming (χ^2^_3_=35.6; *P*<.001), and social networking (χ^2^_3_=26.5; *P<*.001). Those who depended on screens for entertainment and social networking had up to 19% more emotional stress and up to 14% more perceptual stress. In contrast, those who relied on screens for work and professional networking had up to 10% higher levels of life satisfaction. Regression models including age, gender, and 4 stress types explained less than 30% of variation in internet use and less than 24% of the likelihood of being screen addicted.

**Conclusions:**

We showed a robust but heterogeneous link between screen dependency and emotional and perceptual stressors that shift the pattern of screen usage toward entertainment and social networking. Our findings underline the potential of using ludic and interactive apps for intervention against stress.

## Introduction

### Background

In *Understanding Media: The Extension of Man* [1] *,* Marshal McLuhan talked about the relation between media and stress by drawing attention to the work of the father of stress biology, Hans Selye, and the author of *The Stress of Life* [2] *.* Hans Selye had shown that the biological response to psychological threats was identical to the one caused by illness or predatory threat [3]. He called this generalized physiological response stress. A long history of research has ensued to show that although stress is an important adaptive response, chronic exposure to stress would cause various health problems [4]. Referring to the psychobiological evidence, McLuhan proposed that by the virtue of its speed in affective and cognitive stimulation, the electronic media could generate pervasive and cascade neurophysiological interactions that are similar to *stress* (as defined by Selye) would impact the brain’s information processing capacity, leading to pleasure, fear, action, and memory. Today, with the ubiquity of mobile, computationally *smart*, globally connected and socially networked media, McLuhan’s concern about the relation between media and stress adaptation becomes more relevant. Indeed, the relation between screens and stress is extensively researched. Researchers either investigate extremely problematic cases, in which screen addiction becomes a chronic stressor due to disruption of social, mental, and physical health, or investigate the role of screens in coping with chronic stress. Given the ubiquity of various forms of information and communication technologies (ICTs), and their high penetration in the industrial societies, many health researchers and industrial developers are exploring ways to innovate media-based interventions that can mitigate stress [5-9]. However, for the digital health applications (also known as telemedicine or eHealth) to be effective, they must first disambiguate and address the growing concerns about potential risks of screen addiction [10-12].

#### Screens and Coping With Psychological Stress

Numerous studies over decades have emphasized the impact of perception, appraisal, and internal and external factors that define an individual’s risk of developing emotional or health problems due to stress. As screens are communication tools, they can alter the perceptual context and the cognitive and emotional appraisal processes through their messaging. To cope with stress successfully, individuals must dynamically change their perceptual and appraisal landscapes in order to activate cognitive and behavioral adaptations needed to control their stress [13-16]. Stress adaptation is a contextual process and individuals cope with stress through a mix of avoidance-approach [17] or problem- and emotion-focused coping strategies [18], based on their history of exposure and with different resilience factors [19]. In their most popular application, screens are either entertainment devices (that can facilitate avoidance-coping by providing distraction from the source of stress, and relaxation, through endless possibilities for playing games or watching videos), or electronic information centers (that can facilitate information-based and problem-focused approach-coping). The informatics and hypertextual nature of modern screens allows one to personalize them to their coping style according to their cultural or psychological needs. As new screens are also interactive, they not only provide communication and community but also facilitate anonymity and protection from the risks and anxieties of actual encounters. Mobile and internet-connected screens bridge temporal and spatial distances and extend possibilities of seeking support from social and professional networks in addressing specific or general problems. Portable and connected screens (laptops, tablets, and mobile phones) provide a wide range of search tools, production and computation software, and entertainment and playful options that extend an individual’s sense of control not only over space and time but also across emotional and cognitive domains. As such, screens can also help with problem-focused coping to enhance confidence, control, or outcome prediction, for example, by extending one’s functional resources through instantaneous and almost ubiquitous educational, information, communication, and computational resources.

It has been shown that following a major life event, about 57% of a general population (in the Netherlands, with 94% internet penetration) would use the internet for coping, specifically by playing games for mental disengagement and searching information [20]. Communication via blogging has been shown to reduce stress by increasing possibilities for emotion-focused and problem-focused coping through social support [21]. Internet-supported educational or health care interventions are viable for treating stressful physical and mental health conditions [22-31]. For older adults, who face a number of stressors including the loss of cognitive and executive agility, reduced mobility, and diminished social interaction, the opportunity to play video games [32-34] or to engage via online social networks [35,36] has shown positive cognitive and emotional benefits. A systemic review of over 5400 studies of mental health apps on *smartphones* (ie, mobile phones with augmented processing units, with a touch-screen, able to connect to the internet and equipped with accessories such as cameras, voice recorders, etc) suggests promising potential for this mode of intervention in depression and anxiety disorders [37]. There is even experimental evidence to show that being connected to social media can mitigate the physiological response to a psychosocially stressful condition [38] or that adding social media interventions may increase the therapeutic efficacy of pharmacological interventions in treatment of depression [39].

#### Stress-Related Risk of Screen Addiction

If screens can help an individual cope with stress, then it is also plausible that chronic stress would increase the risk of developing neurobiologically consequential screen addiction [40]. The earliest clinical studies of screen addiction go back to the television era [41,42], followed by computer and video games [43], the internet [44-46], and more recently, mobile phone [47]. The target for the majority of these studies is young children or adolescents, or individuals, who suffer a quantifiable disruption in normal life domains (eg, health, finance, family, social relations, and work) as a result of compulsive usage of one technology. These studies underline the correlation between screen usage and stress-related psychopathology [48,49], or the negative health impacts of addiction to television [41,50], computer games [51,52], the internet [53,54], and social media [55-57]. Significant associations between problematic screen use and stressors such as familial instability [58-60] and parental styles [61,62], socioeconomic status and work load [63], have been reported. In a 1-year cohort study of more than 4160 young adults, moderate to excessive computer usage was associated with sleep disturbance in both men and women—whereas greater email/chat usage was correlated with greater risks of mental health problems in women, it was associated with lower perceived stress in men [64]. However, similar studies in the older and nonclinical population are still rare.

### The Research Question

One of the current shortcomings in our knowledge of the relation between screens and stress is that the clinical classifications of screen addiction generally draw on 6 quantifying factors used to diagnose drug dependency: salience, tolerance, withdrawal, interpersonal conflicts, mood alterations, and relapse. However, it has been shown that a general clinical criterion of internet or gaming addiction ignores significant heterogeneity in the accessibility and the content of the medium to which one becomes addicted [65]. Variations in gender and age in terms of vulnerability to stress-related screen usage and self-evaluation of addiction are also important considerations [66,67].

A similar limitation exists in quantifying stress. There are numerous psychometric scales that estimate the risk of being stressed by considering combinations of the emotional and autonomic experience of distress, for example, perceived threats and anxiety [68,69], or life satisfaction [70], and perceptions of self-efficacy and control [71]. Although these questionnaires have common components that underline the stress psychobiology, they do not account for many individual or societal factors that influence the subjective stressfulness of a situation and modulate the functional reserves that are available to the individual for coping with daily stress. Although the clinical questionnaires are designed to be sensitive enough to diagnose the *problematic* or at-risk cases, they may not be sensitive to detecting subtle interindividual heterogeneities that explain variations in general daily screen usage for dealing with normal stressors of life. In the same vein, although there are strong objective markers to link addiction to neurobiology, the less explored individual and socio-relational components may better explain the likelihood of developing stress-related addictions to both drug and certain behaviors—eating, gambling, compulsive internet use, etc [72]. In fact, some argue against the pathological conceptualization of addiction as a purely biological phenomenon and emphasize the primacy of the individual’s choice in seeking pleasure through repetition of a behavior [73].

The aim of this study is to explore the question of stress and screen addiction in a multi-factorial mixed-method fashion that allows us to examine the complexity of stress-related screen dependency.

### Research Approach

In studying the behavioral and contextual differences in usage of communication technologies, media scholars suggest a repertoire-oriented framework that emphasizes the interrelation between different available technologies and underline the importance of characterizing the individual’s choice in the amount of use of different media or content [74]. Existing studies of screen addiction narrowly focus on extreme abnormalities by comparing stratified demographics in relation with specific addictions (eg, gaming, gambling, social networking, and compulsive internet use) and specific clinical manifestations (eg, violence, attention deficit, depression, and anxiety disorders). The repertoires-oriented framework acknowledges the user’s choice between different technologies and in the context of our research asks to what extent would the explanatory factors (in our case, stress or addiction) influence the *pattern* s of different screen uses? This pattern approach is particularly useful in studying the heterogeneity of screen usage arising from subjective versus objective assessments of stress or addiction. It has been shown that the *objective* quantification of stress (be it in terms of socioeconomic, psychometric, or other ratings) does not necessarily correspond to subjective perception of stress [75-79]. Similarly, the majority of definitions of addiction converge on the following elements: hedonic experience following engagement in the behavior, preoccupation with the behavior, loss of control, and suffering negative consequences as a result of losing oneself in the behavior [80]. Engaging in excessive computer use (for research, work, communication, playing, or relaxing) is not necessarily perceived as an addiction to those who engage in the activity. Are there common emotional, perceptual, health, and life domain stressors that distinguish those who consider themselves screen addicted? Do self-described screen addicts have higher scores of internet addiction and screen time? Do they differ from nonaddicts in evaluation of the importance of, access to, and dependence on different screen activities? And finally, are there subtypes of stress that would explain the self-rated screen addiction or the dependence on a given application of screens?

In this study, we have taken a repertoire-oriented approach [74] to explore the relation between stress and patterns of screen usage based on the individual’s subjective assessment of stress and self-rated degree of screen addiction. We hypothesized that individuals who consider themselves screen addicted have higher stress levels than the nonaddicted and that there is a correlation between different types of stress and different types of screen usage to suggest individualized approaches for coping with stress via ICTs.

## Methods

### Survey Design and Distribution

This survey study was conducted in the context of our media-health research, which focuses on designing personalized ICTs for coping with chronic mental and physical health problems. We invited participants to complete an anonymous online survey investigating the relation between screen addiction and health. The multifactorial survey included direct categorical self-assessment questions, as well as indirectly measured scales, to compare the estimated severity of problems (ie, health, screen usage, and stress) versus the individual’s self-categorizations (stressed/not stressed; addicted/not addicted).

The minimum sample size of 355 was determined based on an expectation of 95% confidence level (5% margin of error) in receiving survey responses in a population of 2600—the size of subscribers to the PERFORM Centre’s newsletters and email list of volunteers interested in studying the relation between lifestyle and health. The survey was provided in both French and English. We obtained institutional ethics approval for this study from Concordia University. All participants provided consent, and their participation was fully anonymous and with no remuneration.

The survey obtained demographic information (age, sex, ethnicity, years of education, and profession); Likert-scaled questions about the amount of usage of, dependence on, and importance of different screen types and related activities; and finally, questionnaires to assess vulnerability to different types of stress (details below).

### Screen Variables

#### Screen Addiction

Participants were asked to report if they considered themselves addicted to *computer games or the internet*. If they responded *heavily* or *moderately*, they were categorized into self-admitting screen addicted referred to as *screen addicted (A1*), and if they responded *No*, they were categorized as *nonaddicted (A0)*. We also asked them to estimate the hours (less than 1 hour, 2 to 3 hours, 4 to 5 hours, more than 5 hours) they spent each day on screen-related leisure activities (television, internet, games, and watching videos on computer) to ensure the consistency of self-reported addiction and actual screen time. In addition, we administered a subset of Young’s Internet Addiction test (IAT) [81] including the following items: (1) surfing the internet longer than you intended; (2) forgetting house chores while online; (3) loss of sleep due to internet activities; (4) more time spent online than with family; (5) work or grades suffering as a result of online activities; (6) defensive or secretiveness about being online; (7) nervousness and moodiness due to being offline; (8) preferring online activities over going out; (9) forming new relationships with fellow online users; and (10) others complaining about the amount of time spent online. Each question was scored on a 1 to 5 Likert scale (Never, Rarely, Sometimes, Frequently, or Always). Cronbach alpha on the selected IAT items was .869. The sum of the scores was used as a scale of *internet overuse.*

#### Screen Repertoires

In this report, *screen-repertoires* include electronic display surfaces on which visual content is projected or reflected (eg, a television set, a computer terminal, or a handheld electronic device, such as a tablet or a smartphone) and used for any of the following functions: generation or consumption of information, communication, or entertainment. To investigate the patterns of screen usage, we asked 3 sets of questions. To investigate *How* they use them (*Screen Importance*), they were asked to rate the importance of the following functions in their daily lives: (1) short message service (SMS) or email, (2) playing, (3) online shopping, (4) social networking, (5) searching for information, (6) following the news, (7) watching videos and movies, and (8) e-reading. To understand *Why* individuals use screens (*Screen Dependence*), they were asked to score their daily dependence on screens for the following needs: (1) education, (2) information, (3) entertainment, (4) relaxation, (5) social networking, (6) professional networking, and (7) work. Finally, to assess *What Technologies* they depend on (*Screen Necessity*), they were asked to indicate which technologies they needed to have access to on a weekend or during their vacation: (1) desktop, (2) laptop, (3) smartphone, (4) tablet, (5) e-reader, (6) television, and (7) game console. All questions were scored from 5-point Likert scales (strongly agree to strongly disagree) and were binarized to *High* for agree and strongly agree and *Low* for indifferent, disagree, and strongly disagree responses.

### Stress Variables

#### Working Definition of Stress

Our working definition of stress draws from Mason’s 1968 [82] and Dickerson and Kemeney’s meta-analysis [83] that showed the perception of loss of control in presence of real or perceived self-threatening or unpredictable situations to be the common denominators of triggering a physiological stress reaction. The reason why we focus on this neurological definition is because we are interested in identifying technologies whose impact on stress can be empirically and quantitatively examined in the future. However, instead of focusing on a single stress questionnaire, we investigated 4 potential factors that are likely to be stressful: emotional stress (ES; presence of negative feelings), perceptual stress (anticipation of stressful loss of control and status in common life experiences), health stress (inability to perform normal daily functions), and life domain satisfaction (satisfaction with work, family, social support, finances, and leisure). Internal consistency of the questionnaires was established using reliability analysis. All scores were computed by summing up the Likert scores as described below. The final stress level was computed for each stressor as the percentage of the maximum possible score (ie, if someone expressed highest level of stress in responding to all questions). These ratio scores enabled us to conduct a relative comparison of different stressors’ intensities.

#### Emotional Stress

Emotional stress refers to the state of a personal experience of negative mood and affect such as anxiety, anger, lack of motivation, sadness, or irritability. These mood states can be considered as internal risk factors that explain the interindividual vulnerability to stress. Individuals with mood and anxiety disorders are more stressed [84,85] and are at higher risk of negative health consequences as a result of chronic stress [16,86,87]. We estimated ES using a 5-item questionnaire, adapted from the Depression, Anxiety, and Stress Scale [69], asking participants to rate the following question: *During the past four weeks, how much have you been bothered by any emotional problems such as anxiety, sadness, lacking motivation, being sensitive and irritable, and anger* (scored on a Likert scale 0-3, Not at all; A little; Quite a bit; A lot). The Cronbach alpha value of standardized items was .86.

#### Perceptual Stress

Perceptual stress refers to the vulnerability to experiencing lack of control and perceiving a threat to ego while facing the external world. Unlike ES that measures the actual state of negative feelings and affect, perceptual stress reflects anticipation of a stressful experience. In one of the earliest meta-analysis studies of physiological manifestation of stress, Mason showed that the perception of novelty, unpredictability, lack of control, and threat to ego would reliably predict an autonomic and neuroendocrine response [82]. Dickerson and Kemeney’s meta-analysis of 208 acute stress studies confirms that loss of control in time-limited cognitive tasks or public performance under social evaluative pressure is a reliable trigger of stress response [83]. A common questionnaire to measure perceptual stress is the perceived stress scale, which asks explicit questions about the individual’s sense of control, irritability, uncertainty, and feeling stressed over the past month. We approached the question differently and aimed to assess the general degree of vulnerability to being stressed by commonly lived experiences. We aimed to assess interindividual variations in coping with unknown, unpredictable, and *ego*-threatening circumstances such as being in situations where one may lose control and be under time pressure (such as driving and working overtime) or be judged negatively, for example, in a job interview, public speaking, taking an exam, or going on a first date [82,83,88]. We constructed a 12-item questionnaire and asked the participants to rate how stressful they found the following general situations: (1) not having control (lack of control); (2) making decisions that affect you (uncertainty affecting self); (3) making decisions that affect others (uncertainty affecting others); (4) taking an exam (time/performance pressure), (5) being judged negatively (threat to ego); (6) giving a public speech (social evaluative threat); (7) driving (lack of control); (8) being overworked (lack of control); (9) being in a competition (time/performance pressure under social evaluative threat); (10) getting sick (lack of control); (11) going for a job interview (uncertainty affecting self and social evaluative threat); and (12) going on a first date (social evaluative threat). These items were each scored on a 4-point Likert scale (Not stressful at all, Not stressful, Somewhat stressful, Very stressful, and Extremely stressful). The Cronbach alpha value of standardized items was .81.

#### Health Stress

To be suffering from illness or chronic health conditions is a major stressor that is by and large outside an individual’s locus of control. To evaluate whether individuals suffered health stress, we first asked them to rate the general state of their mental and physical health (good, bad, and could be better). We then asked 7 questions adapted from the Medicare Wellness Checkup survey [89] to assess whether they suffered conditions that would reduce their sense of control over normal daily functions of life. We asked participants to rate how often they have been bothered by any of the following conditions: (1) falling or feeling dizzy when standing up; (2) sexual problems; (3) trouble eating well; (4) problem using the telephone; (5) problem using the computer; (6) problem driving; (7) problem reading; and (8) tiredness or fatigue (scored on a Likert scale 0-4: Never, Rarely, Occasionally, Often, Always). The Cronbach alpha value based on standardized items was .729.

#### Life Dissatisfaction

External factors such as family, friends, work conditions, and financial situations are important well-being factors [90] that can moderate the severity of stress. Supportive and satisfactory personal and professional networks can mitigate adverse effects of health or ES. In contrast, financial, professional, and relationship problems (at work and at home) which are outside an individual’s perceptual, emotional, or practical control can burden their ability to maintain control over their own life. We considered work, family, social relationships, financial comfort, and leisure as life domains to have a potentially significant impact on stress levels. A 6-item questionnaire asked participants to rate how satisfied they felt with the following: (1) My boss is friendly and fair; (2) My work and leisure activities are balanced; (3) My family is supportive; (4) My friends are there for me; (5) My life is under control financially; (6) My work and/or studies are enjoyable (scored on a Likert scale of 0-3, ranging from *true* to *not true at all*). The Cronbach alpha value of standardized items was .72.

#### Self-Reported Stress

To examine the correspondence between our stress variables and the individual’s subjective evaluation of stress, we asked them to report whether they have experienced a recent stressful event. Self-reported stress was a binary variable based on a *Yes/No* (hereafter referred to as *stressed/not stressed*) response to *Have you experienced a major stressful episode in the past year?* We then refined the question by asking participants to check which type of stress they had suffered: bereavement, financial hardship, job loss, school exams, chronic health problems, and relationship problems. These stressors, in terms of their psychophysiological impact, are not equivalent; however, we wanted to capture the heterogeneity in the subjective perception of stress and to compare the intensity of our quantitative metrics in relation to these major life stressors.

### Statistical Analysis

Statistical analyses and visualizations were performed with SPSS 24.0 (IBM, SPSS Statistics, for OX) and Prism 7.0 (Graphpad Inc, for OX).

Univariate statistics were presented as percentages of the response frequencies. Two-way analysis of variance (ANOVA) was used to assess the effect of subjective addiction and stress interaction on screen usage and stress scores. The Kruskal-Wallis test was performed to compare group differences in rating the screen importance, dependence, and access. A posthoc *t* test was used (with Welch-Satterthwaite correction to adjust degrees of freedom for cases where equality of variance was violated) to compare differences in stress scores in relation to rating dependence on different screen-related activities: high or low. Finally, to examine the best model that explained the likelihood of belonging to the screen addicted group, we performed a logistic regression (including different stress scores, age, and gender as explanatory variables). We also tested the same model factors in a regression model with internet overuse as a dependent variable. Statistical significance was set at .05. A test of collinearity was performed to ensure that the variance inflation factor (VIF) was below 3.

## Results

### Sample Characteristics

Out of 654 responders, the final sample size (based on complete case of all variables of interest, that is, screen addiction and stress scores) was 459. Sample characteristics are presented in Table 1. See Multimedia Appendix 1 for more details on the intensity of stress in each category.

### Group Differences in Stress and Screen Usage Scores

**Figure 1:**
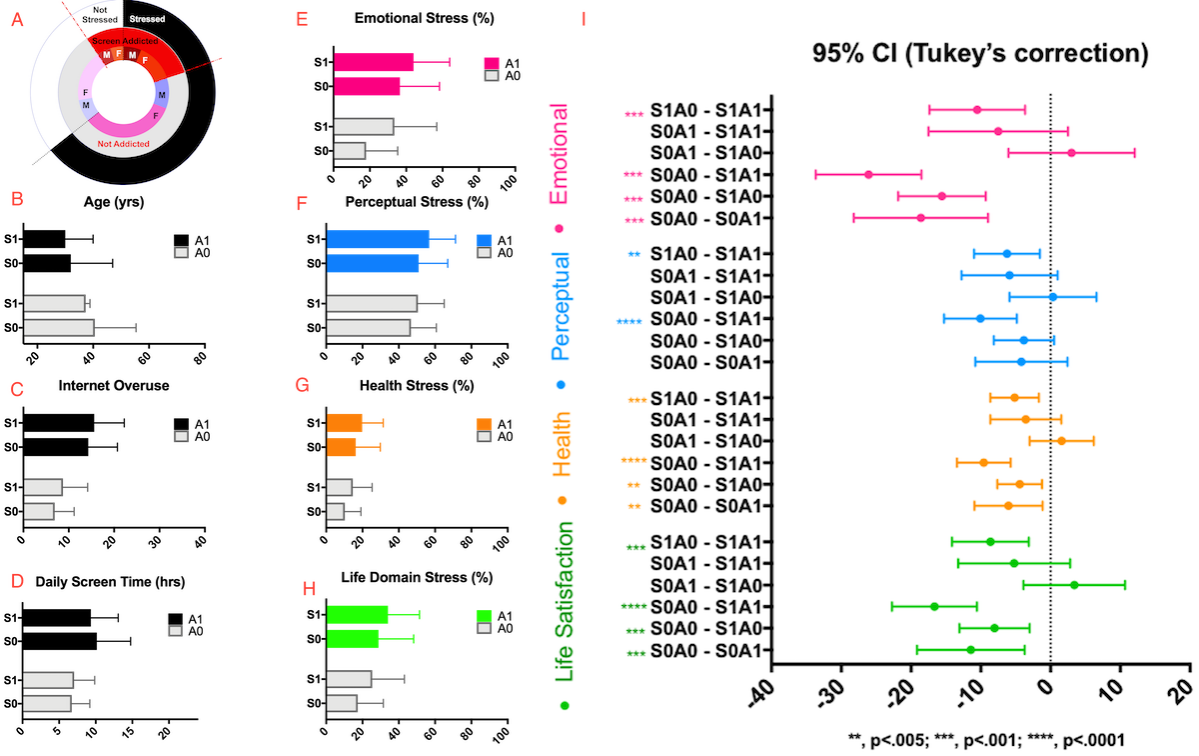
(A) Distribution of the sample based on self-rated screen addiction and recent stress; (B) Group differences in age; (C) Group differences in internet overuse; (D) Group differences in daily screen time; (E) Group differences in emotional stress; (F) Group differences in perceptual stress; (G) Group differences in health stress; (H) Group differences in life dissatisfaction; (I) Posthoc estimated mean differences of stress based on Screen x Addiction categories. The largest differences are observed in comparison of S0A0 (neither addicted nor stressed) versus S1A1 (both addicted and stressed).

Figure 1 (A) illustrates the overlaps in subjective evaluations of stress and screen addiction. Approximately, 30% of the sample considered themselves screen addicted. The majority of the sample reported recent stress but no addiction S1A0 (44.6%, 205/459), followed by S0A0 (25.9%, 119/459), S1A1 (19.8%, 91/459), and S0A1 (9.5%, 44/459). Figure 1 (B) summarizes the ANOVA results. There was no stress by addiction interaction effect on any of the variables (F_1,455_<3, *P*>.1). Individuals who reported themselves as stressed differed in (F_1,455_=5.98, *P*=.02 emotional (F_1,455_=25.4, *P*<.001), perceptual (F_1,455_=9.49, *P=*.002), health (F_1,455_=11.7, *P*<.001), and life dissatisfaction (F_1,455_=13, *P*<.001), internet overuse (F_1,455_=6.83, *P*<.01) but not screen time. In terms of self-rated screen addiction, groups differed in age (F_1,455_=54.3, *P*<.001), ES (F_1,455_=40.4, *P*<.001), perceptual (F_1,455_=11, *P*<.001), health (F_1.455_=23.1, *P*<.001), and life dissatisfaction (F_1,455_=29.7, *P*<.001), internet overuse (F_1,455_=142, *P*<.001), and screen time (F_1,455_=70.2, *P*<.001). Only 23% of variation in internet overuse and 13% of variation in screen time was explained by self-admitted addiction. With the exception of screen time (where no difference between the stressed and nonstressed was observed), the S0A0 group (ie, those who were not stressed and not addicted) reported significantly lower stress and screen usage compared with S1A1 (those who were both addicted and stressed; more details in Multimedia Appendix 2).

### Screen Repertoires With Respect to Self-Reported Addiction and Stress

**Figure 2:**
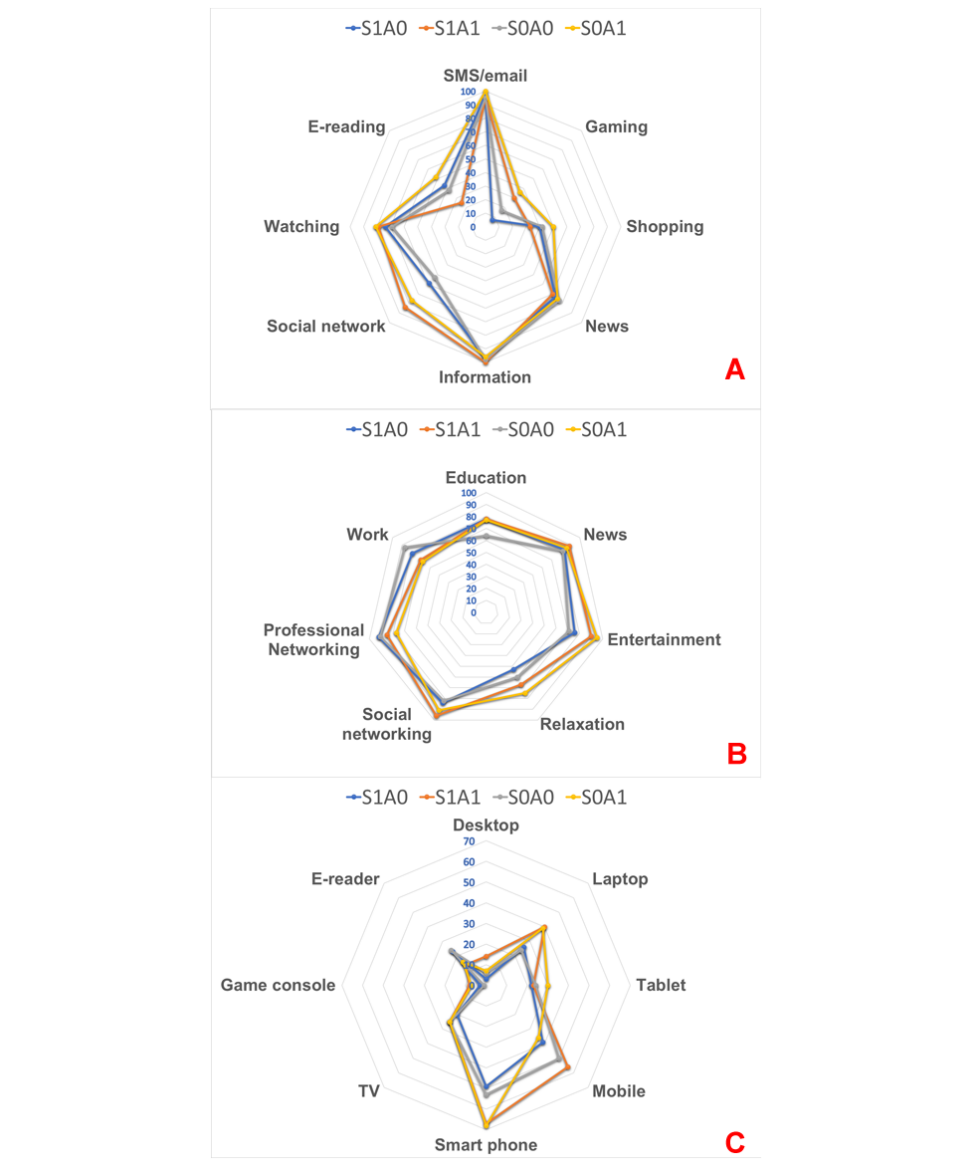
Radar diagram of the ratio of individuals within each group who rated various aspects of the screen repertoires highly; (A) How important are these activities to your daily life? (B) What is the degree of daily dependence on screens for these activities? (C) How essential is it to access these screens on a holiday or during the weekend.

Figure 2 illustrates group differences in the salience of screens in daily life in terms of the frequency of rating a screen or screen-related activity *high*. Results of the Kruskal-Wallis test are presented in Table 2. Significant group differences emerged in rating the importance of daily usage of social networks (highest in S1A1), games (highest in S0A1), and e-books (highest in S0A1). Groups differed in rating the necessity of access to desktop computers (highest in S1A1), laptops (highest in S0, regardless of A), mobile phones (S1A1), smartphones (highest in A1, regardless of S), and game consoles (highest in A1, regardless of S). Groups differed in daily dependency on screens for education (highest in A1, regardless of S), entertainment and relaxation (highest in S0A1), social communications (highest in A1, regardless of S), and professional networking (highest in S1A0).

**Table 1:** Sample characteristics.

Variable		Statistics
**Age (years),** **mean (SD)**		36 (14)
	Younger than 36, n (%)	272 (60)
**Sex, n (%)**		
	Male	136 (30)
	Female	323 (70)
**Education, n (%)**		
	CEGEP	102 (23)
	Bachelor’s	201(44)
	Post graduate	150 (33)
**Ethnicity, n (%)**		
	White	344 (74)
	Hispanic	15 (3)
	Black	22 (5)
	Asian	27 (6)
	Middle Eastern	34 (7)
	Other	16 (4)
**Profession, n (%)**		
	Student	122 (26)
	Educator	58 (13)
	Office professional	50 (11)
	Wellness and health care	48 (11)
	Artist	46 (10)
	Other	135 (29)
**Stressed, n (%)**		
	No	185 (40)
	Bereavement	64 (14)
	Financial	120 (26)
	Exam	107 (23)
	Chronic health	69 (15)
	Relationship	135 (29)
**Mental health, n (%)**		
	Good	272 (60)
	Bad	14 (3)
	Could be better	170 (37)
**Physical health, n (%)**		
	Good	277 (61)
	Bad	15 (3)
	Could be better	167 (36)

**Table 2:** Results of the Kruskal-Wallis test. Percentages of individuals in each category rating each item high or very high (N_High_) are listed (S0, no recent stress; S1, reported recent stress; A0, not addicted; A1, screen-addicted [self-rating]).

Screen-related ratings per groups		S0A1	S0A0	S1A1	S1A0	Χ^2^, *df*=3	*P* value
		N_High_/44 (%)	N_High_/119 (%)	N_High_/91 (%)	N_High_/205 (%)		
**Screen dependence**							
	Education	34 (77.3)	76 (63.9)	71 (78.0)	160 (78.0)	9.1	.03
	News and info	38 (86.4)	98 (82.4)	81 (89.0)	173 (83.9)	3.85	.27
	Entertainment	42 (95.5)	84 (70.6)	82 (90.1)	155 (75.6)	20.5	<.001
	Relaxation	33 (75.0)	71 (59.6)	61 (67.0)	109 (53.2)	9.9	.02
	Social networking	40 (90.9)	97 (81.5)	87 (95.6)	172 (83.9)	10.7	.01
	Professional communications	34 (77.3)	108 (90.8)	77 (84.6)	188 (91.7)	9.6	.02
	Work	30 (68.2)	103 (86.6)	64 (70.3)	163 (79.5)	11.02	.01
**Screen importance**							
	Short message service (SMS) or email	44 (100)	113 (95.0)	86 (94.5)	198 (96.6)	2.9	.4
	Game	16 (36.4)	20 (16.8)	27 (29.7)	15 (7.3)	35.6	<.001
	Shopping	22 (50.0)	50 (42)	30 (33.0)	83 (40.5)	3.89	.27
	Following the news	33 (75.0)	92 (77.3)	64 (70.3)	150 (73.2)	1.4	.71
	Searching	42 (95.5)	117 (98.3)	91 (100)	199 (97.1)	3.85	.28
	Social media	34 (77.3)	63 (52.9)	76 (83.5)	122 (59.5)	26.5	<.001
	Watching	36 (81.8)	82 (68.9)	72 (79.1)	152 (74.1)	4.23	.24
	e-reading	23 (52.3)	45 (37.8)	23 (25.3)	88 (42.9)	11.8	.008
**Screen necessity**							
	Desktop computer	3 (7.1)	7 (5.9)	13 (14.3)	7 (3.5)	12.21	.007
	Laptop	17 (38.6)	28 (23.5)	36 (39.6)	52 (25.5)	9.83	.02
	Tablet	13 (30.2)	28 (23.5)	21 (23.3)	46 (22.4)	1.2	.75
	Smartphone	30 (68.2)	62 (53.0)	60 (66.7)	99 (48.8)	11.39	.01
	Mobile phone	16 (36.4)	60 (50.4)	50 (55.6)	80 (39.4)	9.32	.03
	Television	11 (25.0)	29 (24.6)	23 (25.3)	40 (19.5)	1.9	.59
	Game console	3 (6.8)	1 (0.8)	7 (7.7)	5 (2.5)	9.32	.03
	e-reader	7 (15.9)	29 (24.4)	13 (14.4)	46 (22.5)	4.1	.25

### Different Stressors and Different Screen Dependencies

Differences in various stress types and screen overuse were examined based on dependence (low/high) on different screen-related activities. With the exception of professional networking, all other screen activities were associated with higher internet use. Screen time was not different in relation to depending on screens for professional networking or work (Table 3).

Those who highly depended on screens for entertainment and relaxation had significantly greater levels of perceptual stress. The ES was higher in those who depended on screens for entertainment, social networking, and education. In addition to ES, those who depended on screens for social networking also had higher levels of perceptual and health stress. In contrast, those who depended on screens for professional networking had lower scores of life dissatisfaction and no differences in other stressors. In those who depended on screens for work, both life dissatisfaction and perceptual stress scores were lower (Table 4).

### Heterogeneity of Stress Types and Screen Dependencies

Finally, to examine the heterogeneity in subjective perception of stress and how that would relate to variations in screen dependency, we compared groups based on the type of stress that they reported (Table 5). With the exception of bereavement, irrespective of stress type, all stress scores were higher in those who reported suffering stress in the past year. With the exception of those reporting bereavement and chronic health problems, those who reported other types of stress were younger and had greater scores of internet overuse. Differences in stress levels are presented in Multimedia Appendix 1.

**Table 3:** Differences in screen usage based on screen dependence (number of individuals rating screen usage as high [N_high_] vs number of individuals rating screen usage as low [N_low_]).

Dependence	Screen usage	Internet use		Screen time	
	N_high_/N_low_	95% CI	*P* value	95% CI	*P* value
Education	344/18	0.1 to 2.8	.03	0.3 to 1.7	.004
News	389/70	.02 to 3.3	.05	0.1 to 1.8	.03
Entertainment	363/96	2.3 to 5.2	<.001	0.3 to 1.8	.008
Relaxation	274/185	1.5 to 3.8	<.001	0.1 to 1.33	.03
Social networking	396/63	2.6 to 5.9	<.001	0.8 to 2.54	<.001
Professional networking	407/52	—^a^	—	—	—
Work	360/99	−2.0 to 0.72	.005	—	—

^a^No significant difference was observed.

**Table 4:** Differences in stress levels based on screen dependence (number of individuals rating screen usage as high [N_high_] vs number of individuals rating screen usage as low [N_low_]).

Dependence	Screen usage	Emotional		Perceptual		Illness		Life dissatisfaction	
	N_high_/N_low_	95% CI	*P* value	95% CI	*P* value	95% CI	*P* value	95% CI	*P* value
Education	344/18	3.4 to 12.9	<.001	—^a^	—	—	—	0.5 to 5.1	.01
News	389/70	—	—	—	—	—	—	—	—
Entertainment	363/96	1 to 11.3	.01	0.7 to 7.4	.01	—	—	—	—
Relaxation	274/185	—	—	3.6 to 9.1	<.001	—	—	—	—
Social networking	396/63	7.5 to 19.5	<.001	7 to 14.8	<.001	—	—	1.8 to 7.7	.005
Professional networking	407/52	—	—	—	—	−17.5 to −5.4	<.001	—	—
Work	360/99	—	—	−8.2 to −1.5	.005	−9.9 to −0.6	.03	—	—

^a^No significant difference was observed.

**Table 5:** Differences between stressed and nonstressed groups based on the type of stress. Values represent the estimated mean differences between those reporting the type of stress versus others. Stress scores are normalized to the highest possible score for each stress type.

Stress type	Age	Internet overuse	Screen time	Emotion	Perception	Health	Dissatisfaction
Bereavement	—^a^	—	—	—	—	—	—
Financial	−5.8^b^	2.6^b^	—	13.9^b^	4.6^c^	5.14^d^	13.7^d^
Exam	−14.9^b^	3.9^b^	1.06^c^	19.9^b^	10.7^d^	7.4^d^	8.4^d^
Chronic health	—	—	—	9.6^d^	5.4^c^	5.5^d^	8.5^c^
Relationship	−4.17^c^	1.7^c^	0.8^c^	12.2^b^	3.1^c^	3.6^c^	7.5^d^
Mental health	−7.4^d^	3.8^d^	0.9^c^	29.8^b^	10.5^b^	9.7^b^	15.3^b^
Physical health	−2.9^c^	3.4^b^	—	15.3^b^	6.5^b^	8.6^b^	11.2^b^

^a^No significant difference was observed.

^b^*P*<.001.

^c^*P*<.05.

^d^*P*<.01.

**Table 6:** Parameter estimates for logistic regression, dependent variable: screen addiction.

Model A	Regression coefficients (B)	Standard error	Odds ratio (95% CI)	*P* value
Age	−.039	0.010	0.962 (0.944 to 0.980)	<.001
Emotional stress	.005	0.006	1.005 (0.993 to 1.017)	.43
Perceptual stress	.015	0.009	1.015 (0.997 to 1.032)	.09
Health stress	.022	0.012	1.022 (0.999 to 1.045)	.07
Life dissatisfaction	−.023	0.006	0.978 (0.967 to 0.989)	<.001
Sex (F<M)	.898	0.258	2.454 (1.47 to 4.07)	.001

**Table 7:** Parameter estimates for linear regression, dependent variable: internet overuse.

Model B	Standardized coefficients (B)	Standard error	*t* value	95% CI for B	*P* value
Age	−0.223	0.019	−5.28	−0.137 to −0.063	<.001
Emotional stress	0.165	0.015	3.14	0.017 to 0.075	.002
Perceptual stress	0.139	0.020	3.04	0.021 to 0.098	.002
Health stress	0.111	0.027	2.34	0.010 to 0.117	.02
Life dissatisfaction	0.200	0.013	4.62	0.035 to 0.087	<.001
Sex (F<M)	−0.147	0.581	−3.56	−3.2 to −0.913	<.001

We found significant differences in dependence on social networks in individuals who reported financial, exam relationship, and mental health stresses compared with the nonstressed, those who reported exam stress were more dependent on screens for education, entertainment, relaxation, and social networking but less for work. To control for the sample bias, we reran the analyses on 188 samples, after excluding all students. In this case, the only significant difference in screen dependency was in social networking (higher in those with financial stress; *t*_186_*=2.7, P*<.01)*.* In this subgroup, age, internet usage, and screen time were not dependent on stress, but robust differences in stress scores (ie, those with *P*<.001) were also observed in this subgroup.

### Regression Analysis of Age, Gender, and Stress in Relation to Subjective Screen Addiction and Quantitative Internet Overuse

Having identified group heterogeneities both in terms of different stressors and different screen-related activities, we investigated to what extent variables such as age, gender, and various stress factors explained the likelihood of being screen addicted (logistic regression, Model A, see Table 6) and internet overuse (linear regression, Model B, see Table 7). The VIF for independent variables was below 1.5, thus the model had sufficient tolerance to collinearity. Cross-correlation coefficients are provided in Multimedia Appendix 2.

In Model A (which explained 24% of the variance in the likelihood of being in the A1 group), to be younger, male, and have lower life satisfaction were the most important predictors of the likelihood of identifying oneself as screen addicted. In Model B (which explained 30% of variations in internet overuse), all factors were significant, with age being the strongest factor followed by dissatisfaction, ES, gender, perceptual stress, and finally health stress.

## Discussion

### Principal Findings

We examined the relation between screens and stress using the repertoire-oriented media research framework and showed significant associations between self-admitted screen addiction and quantitative stress levels, as well as stress-specific usage of screens. Individuals who consider themselves screen addicted are also more stressed and are more likely to use screens for entertainment and social networking.

An important finding is that the relation between subjective and objective self-assessment of stress and screen addiction is not overlapping. Although 65% of survey responders reported having suffered various stressful events, only one-third of those also considered themselves *screen addicted.* Self-reported stress did not predict significant likelihood of belonging to the screen-addicted group either. However, certain stressors such as financial, relationship, exam, and health problems were associated with higher rates of screen overuse. Interestingly, the magnitude of the estimated difference in stress levels was larger in the self-admitting addicted versus the nonaddicted group, compared with the self-admitting stressed versus nonstressed group, suggesting an implicit link between actual stress and perception of screen addiction. The screen addicted group had significantly higher internet use and screen-time scores, therefore confirming that subjective assessment of screen addiction corresponded to actual usage metrics, but the average scores were not very high; therefore, it is unlikely that any of the participants were problematic screen users.

In addition, we explored differences in the pattern of screen repertoires in different subgroups. The general patterns were similar between all groups, with following the news and gathering information being the highest and equally important activity in all groups. The necessity of access to smartphones was the highest, and the necessity of access to game consoles, followed by television, the lowest. The pattern of daily dependence on various functions was equipotent (above 70%) across all possible activities, but the center of the pattern shifted toward entertainment and relaxation for the self-admitting addicted and toward work and professional networking for the nonaddicted. The strongest pattern differences emerged at the level of the importance of social networking and gaming and dependence on screens for entertainment. In fact, emotional, perceptual, and health stress were significantly higher in those who depended on screens for social networking. Perceptual stress was also higher in individuals who used screens for entertainment and relaxation. In contrast, individuals who used the screens for work had lower perceptual stress and higher life satisfaction. Post hoc analysis of the effect of subtypes of stress on screen dependency further confirmed that social networking was important to those reporting financial, relationship, mental health, and exam stress. The fact that exam stress was the only type of stress to predict differences in dependence on entertainment and relaxation indicates a demographically specific effect exclusive to a younger student subsample. However, after excluding students from the sample, differences in social networking related to financial stress were still significant.

Our regression analyses show that age and gender influence the prevalence of screen dependency, but only a small portion of variations in screen addiction (24%) or internet overuse (30%) was explained by stress and demographic factors; therefore, other variables must contribute to individual’s screen usage patterns.

### Comparison With Previous Work

#### Robust Association Between Stress and Social Networking

In our sample, over 90% of screen addicted and over 80% of the nonaddicted considered social networking highly important, consistent with global statistics, indicating that the percentage of adults using social media has reached 94% [91,92]. Griffith and Szabo have shown that social networking is the most prevalent of all online activities [65]. In our study, variations in social networking emerged as the most robust indicator of reporting oneself as screen addicted, concurrent with having high levels of emotional and perceptual stress—mainly in the young students and also in a subgroup who reported financial stress. In general, the proportion of self-admitting, stressed individuals who rated social networking important was high. Individuals who depended on screens for social networking had larger scores of emotional, perceptual, and life dissatisfaction. These findings corroborate an earlier review of uses and gratifications research that revealed that individual’s dependence on social media related to their need for relationship maintenance, passing time, entertainment, and companionship [93]. It has been argued that through a myriad of stimulation and interaction, social media can modify mood for better or for worse [38,94,95]. Could social networking have caused the higher stress levels? It has been shown that spending time on Facebook causes a decrease in mood by increasing envy and reducing the social capital [48,49], increasing anxiety about relationships [96,97], or increasing guilt about having wasted time [98]. Our cross-sectional study design precludes any conclusions about the causality of the relationships we report, but it is plausible to suggest that those who consider themselves screen addicted perceive time spent on social networks with a more negative connotation, than time spent on following the news or searching information—which were both the most important activities for all groups.

#### Habitual Screen Use for Coping With Stress

We observed a relative shift toward depending on screens for entertainment, relaxation, social networking, and education in the subjectively stressed and screen addicted group (S1A1). This group had approximately 26% greater ES compared with the S0A0. In contrast, dependency on screens in the S0A0 shifted toward work and professional networking. When asked about the importance of a set of activities on a regular basis, in addition to social networking, playing games was important to 36% of S0A1 group, and 31% of S1A1, versus 17% of the S0A0, suggesting that gaming was not related to the experience of stress. Nevertheless, approximately 8% of the S1A1 group considered access to game consoles necessary, as opposed to only 1% of the S0A0. One possible explanation is that using games serves as a coping strategy against anxiety, which is consistent with the observation of significantly higher associations between emotional and perceptual stress and greater dependency on screens for entertainment and relaxation (particularly in students). This interpretation is in line with previously reported comorbidities between anxiety and depression and excessive use of games or internet, mainly in the young [58-64,99,100] This raises the question, should the excessive usage of screens (games and social networks) for relaxation and entertainment be considered as addiction? Is this cultural connotation the reason why those who use screens for leisure activities are more likely to label themselves as screen addicted? Or is it because spending too much time on screens distracts students from the school work and thus becomes a stressor?

There is controversy whether behavioral compulsions should be treated as addiction disorders or as an individual’s adaptive choices [73,101] that should be dealt with by accounting for socio-relational heterogeneities [72]. Weisel et al have previously suggested that screen *addiction* is not necessarily a problematic phenomenon (as it is commonly referred to in the literature), but a manifestation of the individual’s coping strategy, which should be channeled toward care [24]. Despite the evidence that social networks such as Facebook can be stressful [48,49,96-98], there exists some empirical evidence to suggest that being connected to social media can mitigate the physiological response to a psychosocially stressful condition [38] or that adding social media interventions improves the outcome of traditional psychiatric treatment of depression [39]. Games also interact with myriad cognitive, executive, and rewards processes, and as such they have a quantifiable impact on physiological stress response [102,103]. That perceptual stress (measured by questions about the degree of feeling stressed by control and ego-threatening situations) was higher in those who relied on screens for entertainment and relaxation is noteworthy. Recall that we defined stress as the body’s adaptive response to restore mental or physiological balance while challenged by external or internal, perceived or real, threats to self [88]. This biological framework is important in interpretation of screen-related stress, because the inverted U-shape of stress response determines which kind of activity causes or diminishes the physiological response. In dealing with stress, individuals adopt problem-focused or emotion-focused coping styles, that can be based on either avoidance or approach to confront a stressor [13,18]. To play games for mental disengagement following a major life event may be a form of problem-focused coping [20].

Should we rethink the generally negative connotation in linking stress to screen-*addiction*? In our sample, approximately 70% the S0A0 depended on screens for entertainment, and the highest ratio of dependence on screen for entertainment (95%) was S0A1, that is, those who considered themselves screen addicted but not stressed. Recall that despite the fact that stress levels were high in screen addicted individuals, stress, age, and gender explained no more than 30% of variations in internet overuse and no more than 24% of the likelihood of being in the group who identified themselves as screen addicted. Also, recall that to use screens for work, information and news were the most salient of activities for everyone, but they did not differ across groups. Therefore, it is perhaps not the amount of screen usage, but subjective differences in justification of using screens for leisure activities that explain our results. Future studies are needed to explore these questions in relation to personality and perceptual factors.

### New Contributions

#### Subjective Rating of Screen Addiction in Relation to Different Types of Stress

To the best of our knowledge, this is the first study to examine the relation between subjective assessment of screen addiction, various sources of stress, and various screen-related activities. Approximately, one-third of our sample identified themselves as screen addicted, and indeed the scores of internet overuse and screen time in this group were significantly greater than the nonaddicted. Although it should be noted that the scores were not near the maximum, suggesting that the self-assessments reflected a personal perception of inadequate screen usage, rather than an actual *abuse*. To have accounted for both subjective and quantitative measures of stress and addiction revealed interesting differences in the magnitude of stress based on stress category versus addiction category. Interestingly, self-admitted screen addiction revealed greater difference in all stress categories, compared with reporting recent stress. To account for this subjective difference is important particularly in the context of studying the relation between stress and screen use.

We took a similar multivariate approach to stress as well and found that serious stressors such as bereavement and chronic health were not associated with differences in internet overuse, screen time, or any daily screen dependency, but financial, exam, relationship, and self-evaluated mental health stressors were associated with greater dependency on social networking. This multifactorial approach helped identify subtle differences in the type of stress-screen association. For instance, we found that dependence on screens for entertainment and social networking was associated with greater emotional and perceptual stress in contrast to dependence on screens for work, which was associated with lower scores of dissatisfaction and smaller perceptual stress scores. Although different stress scores shared some variance (no more than 28%), they were not strongly collinear, and each measured different sources of vulnerability. Emotional stress reflected the impact of existing and prospective feelings and anxieties that are experienced by an individual. Perceptual stress coded interindividual variations in self-confidence to cope with unknown, unpredictable, and *ego*-threatening circumstances such as being in situations where one may be judged negatively, that is, in a job interview, public speaking, taking an exam, or going on a first date [82,83,88]. Life satisfaction related to external factors that include relationships at work and with friends and family, financial control, and work/leisure satisfaction. These findings underline the necessity of designing experiments that account for personal and social variants that account for population heterogeneities in media selectivity, resilience, and coping [72-74].

#### Patterns of Screen Usage in Relation to Different Types of Stress

To the best of our knowledge, this is the first study to have applied the media-repertoire framework and to have studied the interrelations between various screens in relation to stress. Griffiths et al have argued that in studying behavioral dependence on screens, the heterogeneity of activities ranging from news and shopping to gaming, social networking, etc must be accounted for [104]. To evaluate this view, we examined the prevalence of various types of screens (such as TVs, computers, and e-readers and tablets) or screen-related activities such as watching videos and movies, reading, and working, which provide a comparative reference to guide designing better screen technologies and interfaces for stress management. In our sample, we did not observe any group difference in using tablets, TV, or e-readers; nor in activities such as email or SMS, following the news, watching videos, or reading online. The dependence on screen for education or gathering information and news was also not different. Instead, the differences were significant in the importance of gaming and social networking, in dependence on screens for relaxation, entertainment, and social networking, and in the necessity of access to game consoles, mobile and smartphones, and computers on a weekend. These findings suggest that portability, communication, and leisure are important features for those with higher levels of emotional and perceptual stress, thus confirming the potential of digital health mobile apps for mitigating stress through ICTs [105].

### Limitations

Sampling biases in this study confound interpretations. We designed this study to target internet-literate and self-conscious individuals who are concerned about the negative impact of screen-addiction on health. Our snowball survey method produced an age- and sex-biased sample, in which the majority of responders were female, younger than 36 years of age, white, and university-educated. This biased sampling is common in digital surveys and reveals which demographics are more likely to utilize and benefit from ICT in health intervention. Although it limits the generalizability of the conclusions in designing global digital health solutions, it also underlines the potential to work toward creating more inclusive digital ecologies.

These findings should be considered as an exploratory approach to the investigation of the interactions between screens and stress—both highly relevant for public health innovations. However, our data warrant no clinical interpretations. The term *screen addiction* must be interpreted exclusively in the context of subjective self-evaluation. Although we showed that self-admitting screen addicts have significantly greater screen time and internet usage, our survey is limited in revealing dimensions of salience, tolerance, and emotional dependence to screens. It is also limited in explaining the relation between screen usage and health states, which would be necessary for clinical categorization of a behavioral addiction.

Finally, no inferences about the causality of the relationships between stress and screen use are justified. Instead, we emphasize the heterogeneity of stress-related factors that can moderate screen-related behaviors. Our findings underline the importance of multivariate examination of screen dependency within various psychological or sociological context. Future studies are needed to explore socioeconomic and intergenerational variations more closely.

### Conclusions

As McLuhan predicted, electronic media (television, at the time) is *an extension of our physical bodies*, which interacts with our adaptation system to restore our physical and psychological equilibriums. Our interdisciplinary approach provided evidence for the contextual heterogeneity of the relation between screens and their role in stress adaptation, specifically via online activities for entertainment and social networking. Future work needs to examine the clinical implications of these findings and explore the mediating effects of a screen-related lifestyle on mental health outcomes.

## Appendix

Multimedia Appendix 1 Differences in stress levels and screen addiction with respect to the type of major stress suffered in the previous year.

Multimedia Appendix 2 Tests of correlation and collinearity between different stress and screen variables.

## Abbreviations

ANOVA: analysis of variance
ES: emotional stress
IAT: Internet Addiction test
ICTs: information and communication technologies
SMS: short message service
VIF: variance inflation factor
