# Supplementary material for: To Each Stress Its Own Screen: A Cross-Sectional Survey of the Patterns of Stress and Various Screen Uses in Relation to Self-Admitted Screen Addiction
Source: J Med Internet Res. 2019 Apr 2;21(4):e11485. doi: 10.2196/11485 (PMC6465981; doi:10.2196/11485)

Appendix 1: Variations in stress levels depending on the type of stress suffered in the past year.

Stress is a heterogeneous phenomenon, and therefore, in stressed individuals, we asked them to identify the type of stress they had suffered. In the figure below, we demonstrate variations in stress-levels depending on the reported stress types.

Recall that logistic regression with self-described stress as predictor did not reveal a significant likelihood of being screen addicted. This can be seen in the absence of a significant difference in internet overuse, and in screen time as well.

Stress Levels for Reported Stress Types

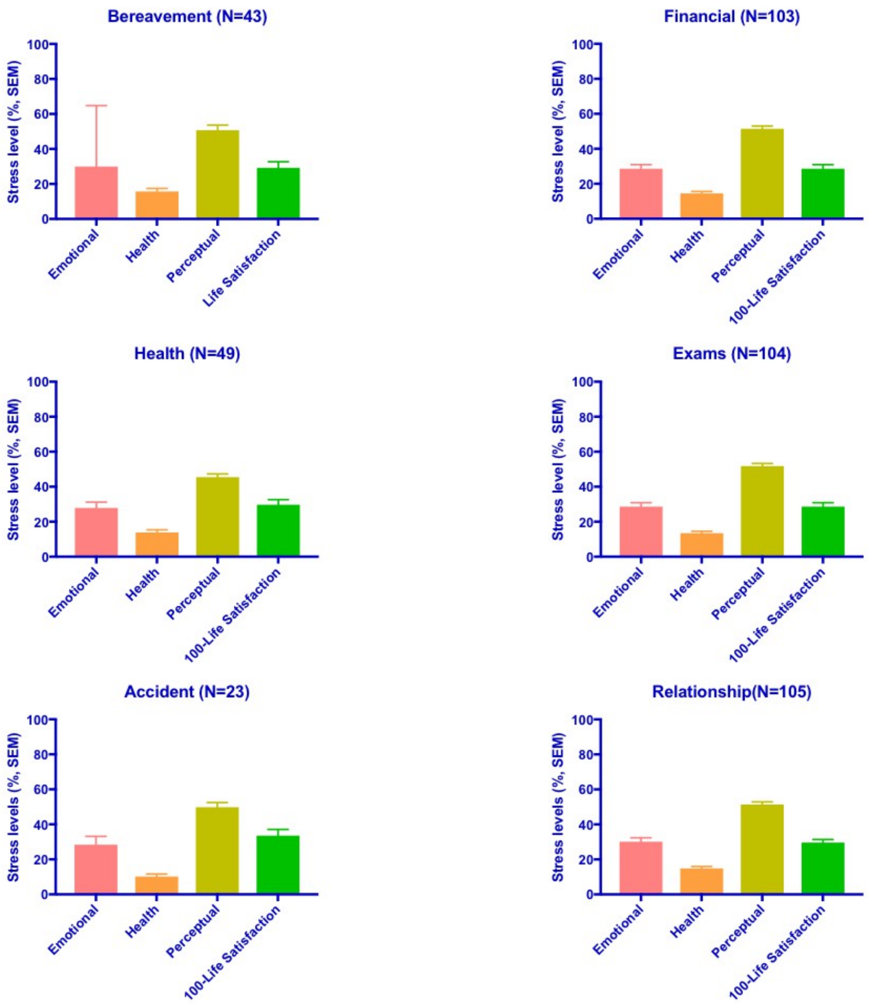

Screen Usage Depending on Stress Type

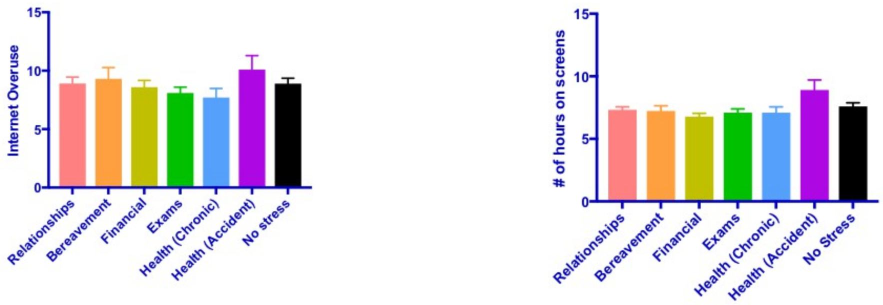

Supplement: Multimedia Appendix 1 [file jmir_v21i4e11485_app1.pdf]
