# Supplementary material for: To Each Stress Its Own Screen: A Cross-Sectional Survey of the Patterns of Stress and Various Screen Uses in Relation to Self-Admitted Screen Addiction
Source: J Med Internet Res. 2019 Apr 2;21(4):e11485. doi: 10.2196/11485 (PMC6465981; doi:10.2196/11485)
Supplement: Multimedia Appendix 2 [file jmir_v21i4e11485_app2.pdf]

Appendix 2:

1. Cross Correlation Table

Correlations

	Age	Internet Use	Screen time	emotional	perceptual	health	dissatisfaction
Age	1						
Internet Use	-.340**	1					
Screen Time	-.294**	.443**	1				
Emotional Stress	-.334**	.430**	.198**	1			
Perceptual Stress	-.217**	.295**	.221**	.425**	1		
Health stress	-.240**	.331**	.125**	.531**	.339**	1	
Life dissatisfaction	-.041	.335**	.122**	.404**	.205**	.269**	1

**. (Pearson's) Correlation is significant at the 0.001 level (2-tailed).

2. Test of Collinearity

Coefficients[a]

Model		Correlations			Collinearity Statistics	
		Zero-order	Partial	Part	Tolerance	VIF
1	Age	-.340	-.340	-.340	1.000	1.000
2	Age	-.340	-.244	-.219	.913	1.095
	Screen Time	.443	.382	.359	.913	1.095
3	Age	-.340	-.147	-.121	.834	1.199
	Screen Time	.443	.369	.324	.902	1.108
	Emotional	.430	.343	.298	.877	1.140
4	Age	-.340	-.139	-.113	.829	1.206
	Screen Time	.443	.371	.324	.902	1.108
	Emotional	.430	.250	.209	.667	1.500
	Health	.331	.127	.103	.714	1.401
5	Age	-.340	-.136	-.111	.828	1.208
	Screen Time	.443	.362	.314	.885	1.130
	Emotional	.430	.227	.188	.617	1.620
	Health	.331	.117	.096	.699	1.430
	Perceptual	.295	.060	.048	.782	1.280
6	Age	-.340	-.165	-.132	.814	1.229
	Screen Time	.443	.356	.301	.880	1.136
	Emotional	.430	.158	.127	.557	1.795
	Health	.331	.104	.083	.695	1.438
	Perceptual	.295	.055	.044	.781	1.281
	Life dissatisfaction	.335	.206	.166	.816	1.226

a. Dependent Variable: Internet Overuse

3. Stress by Addiction Interactions

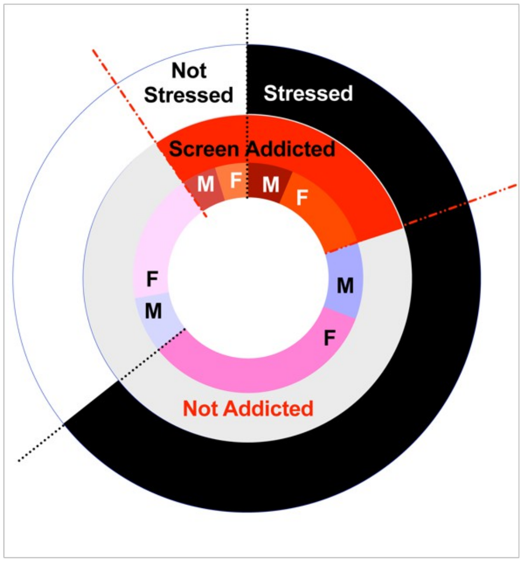

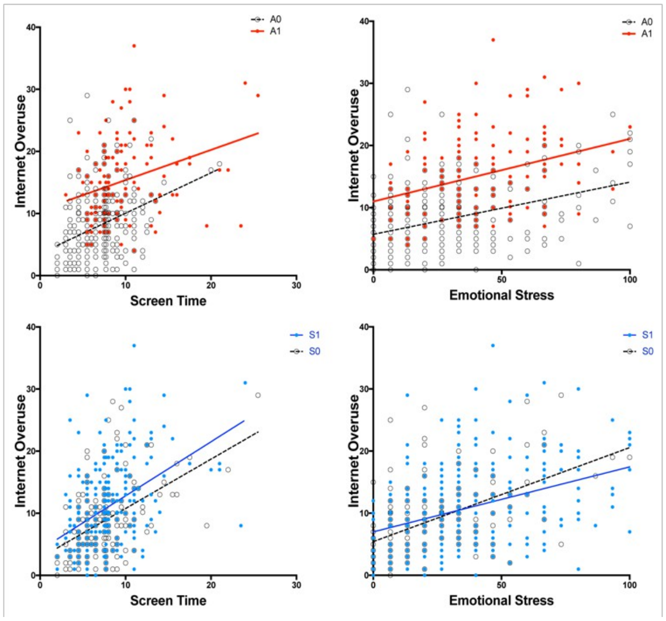

Regression analysis:

	A0	A1	S1	S0
Overuse/screen time	0.6503 ± 0.09949	0.4861 ± 0.1324	0.871 ± 0.1056	0.7948 ± 0.1156
	Slopes are not different: F(1,455)=1.1, p=0.29<br>Intercepts are different: F(1,455)=94, P<.0001		Slopes are not different: F(1,455)=0.22, p=0.63<br>Intercepts are different F(1,455)=11.1, P=.0009	
Overuse/Emotional	0.08399 ± 0.01174	0.1008 ± 0.02555	0.1046 ± 0.01581	0.1514 ± 0.01931
	Slopes are not different: F(1,455)=0.4, p=0.50<br>Intercepts are different: F(1,455)=113.9, P<.0001		Slopes are not different: F(1,455)=3.0, p=0.08<br>Intercepts are not different: F(1,455)=0.2, P=.61	
